# The Comparison of A New Durable Coronaplasty Technique with Norfolk Method for Glans Reconstruction after Phalloplasty

**DOI:** 10.29252/wjps.9.1.39

**Published:** 2020-01

**Authors:** Mohammad Reza Akhoondinasab, Mahdy Saboury, Yousef Shafaeei, Siamak Forghani, Mohammad Javad Fatemi

**Affiliations:** Department of Plastic and Reconstructive Surgery, St. Fatima Hospital, School of Medicine, Iran University of Medical Sciences, Tehran, Iran

**Keywords:** Transsexual, Phalloplasty, Coronaplasty, Norfolk

## Abstract

**BACKGROUND:**

Phalloplasty is the most amazing reconstructive surgery, and has a vital role in the quality of life of transsexual patients. There are several techniques for glans sculpting, but none of them had long-lasting results. In the present study, a new technique was introduced and compared with Norfolk technique for coronaplasty following phalloplasty.

**METHODS:**

In the present randomized controlled study, 40 transgender patients were enrolled from February 2016 to December 2018, at St. Fatima Plastic and Reconstructive Surgery Center. The patients were randomly assigned in two groups including 20 patients with anterolateral thigh flap (ALT)/radial forearm free flap (RFFF) phalloplasty followed with our new coronaplasty technique (group 1) and 20 patients with ALT flap/RFFF phalloplasty followed with Norfolk technique (group 2).

**RESULTS:**

Almost 85% of the patients underwent the surgery with the new technique were satisfied with the outcome of surgery and considered it acceptable within 6-month follow-up, however, only 70% of the patients in Norfolk technique group reported acceptable results, which was significantly lower than the new technique. Similarly, within 12-month follow-up, 80 and 40% of the patients, respectively in new and Norfolk groups reported acceptable results, which was also significantly higher in the new technique.

**CONCLUSION:**

This new technique showed remarkably better results relative to the usual technique for glans sculpting in transsexual patients. Moreover, it had the ability to be easily applied along with ALT/RFFF flaps in both immediate and delayed situations.

## INTRODUCTION

Phalloplasty is the most amazing reconstructive surgery and its vital role in the quality of life of the transsexual patients is undeniable. There are several techniques used for glans sculpting, but none of them have long-lasting results. Flattening of the coronal ridge is one the most common outcomes (1). As the greatest plastic and reconstructive surgery center in Middle East, we perform at least 10 sex reassignment surgeries in a month. Therefore, we have an invaluable dataset for this kind of operation. We often apply two common phalloplasty techniques including Radial Forearm Free Flap (RFFF) and pedicled Anterolateral Thigh flap (ALT) at our center. Recently, the introduction of novel techniques such as ALT FLAP/RFFF and functional prosthesis has not only improved the surgical outcome but also aesthetical aspects of this procedure (1). Thus, in the present study, a new technique was introduced and compared with Norfolk technique for coronaplasty following phalloplasty.

## MATERIALS AND METHODS

In the present randomized controlled study, 40 transgender patients were enrolled from February 2016 to December 2018, at St. Fatima Plastic and Reconstructive Surgery Center affiliated to Iran University of Medical Sciences in Tehran, Iran. The exclusion criteria were smoking, predisposing diseases (diabetes mellitus, collagen vascular disease), and inability or refusing to undergo the follow-up. The patients were randomly assigned in two groups including 20 patients with ALT flap/RFFF phalloplasty followed with our new coronaplasty technique (group 1) and 20 patients with ALT flap/RFFF phalloplasty followed with Norfolk technique (group 2). 

**Fig. 1:**
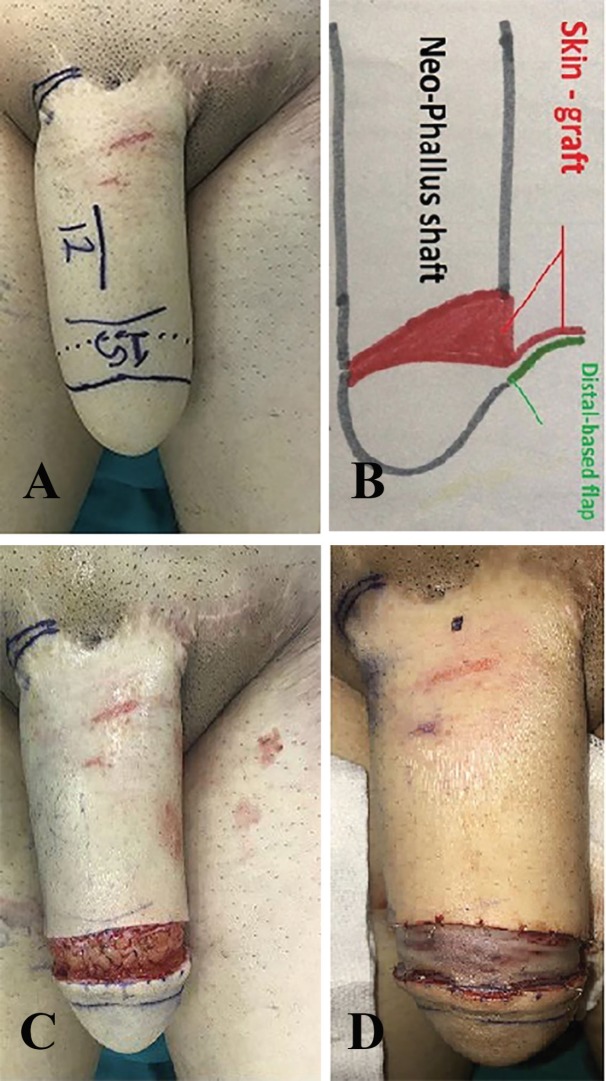
**A:** Marking. **B:** Schematic view of the new technique. C**:** Flap rising. **D:** Medium thickness skin graft covered the donor site and the dorsal aspect of the raised flap

The surgery was performed by the same surgeon for both groups after obtaining an informed consent from all the patients. For patients in group 1, the neo-glans length was considered equal to neo-phallus diameter (Figure 1A). With an oblique (45 degree) incision, distally-based flap (with 1 cm width) was raised to create a neo-glans with greater dorsal length relative to ventral length (Figure 1B and C). Then, medium-thickness skin graft was used to cover the whole defect area. 

**Fig. 2:**
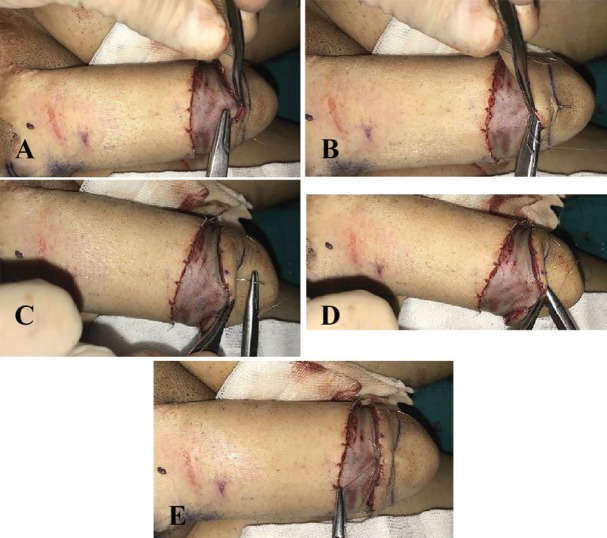
**A:** First needle passage. **B:** Full thickness suture. **C:** Making horizontal mattress suture. **D:** Second needle movement. **E:** Knotting

Skin graft should be crescent-like and wide enough to cover both defect and dorsal areas of raised flap. To perform the procedure, the two ends of skin graft were sutured to the ends of the defect area with adequate tension, and then it was fixed to the distal and proximal edges of the defect area (Figure 1D). To create distinct coronal sulcus and coronal ridge and achieve aesthetically favorable results with naturally appearing glans, 5/0 Monocryl mattress sutures were used through the skin graft and beneath the coronal flap toward the distal end of the neo-glans (Figures 2A-E). 

**Fig. 3:**
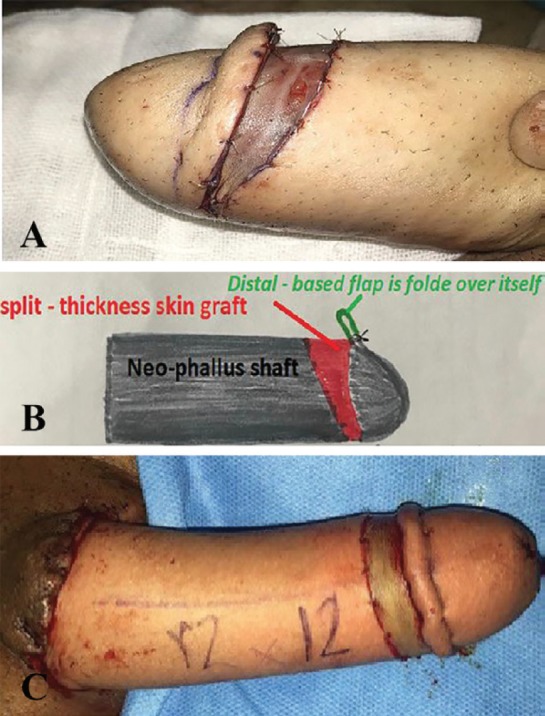
**A:** Final view of new technique. **B:** Schematic view of Norfolk technique. **C:** Final view of Norfolk technique

At the end (Figure 3A), grafted area was covered with petroleum-gauze dressing, which was removed 5 days after the procedure. For patients in group 2, Norfolk technique was performed as following: On the neo-phallus, an 11- cm long incision was made about 3 cm dorsally away from the tip, and was deepened to the subcutaneous tissue (1). Distal undermining from the incision site was carried out for about 1 cm on the dorsal aspect reducing to 0.5 cm on the ventral aspect (Figure 3B). The circumferential skin flap created from undermining was folded over itself and was fixed with mattress sutures (Figure 3C). 

The raw surface was covered with split-thickness skin graft. The grafted area was covered with a tie over petroleum-gauze dressing, which was removed 5 days later. The results were scored subjectively on a 3-point scale by the patient and objectively by an assessor (another plastic surgeon) at 6 and 12 months after the operation. The total score was classified as 1 (unacceptable), 2 (fair), and 3 (acceptable). UMIN Clinical Trials Registry was undertaken as Unique ID issued by UMIN: UMIN000036014 and Receipt number of R000041025 at https://upload.umin.ac.jp/cgi-open-bin/ctr_e/ctr_view.cgi?recptno=R000041025. The statistical analysis was performed using IBM SPSS software (Version 22.0, Chicago, IL, USA), and the *p* value less than 0.05 was considered as statistically significant. 

## RESULTS

At 6-month postoperative follow-up, there was one patient with score 1 (unacceptable) in Norfolk group, whereas such case was not reported in new technique group. On the other hand, there were 5 and 3 patients who scored 2 (fair) in Norfolk and new technique groups, respectively. In addition, 17 patients in new technique group believed that, the results were acceptable, compared to 14 patients in Norfolk group (*p*=0.03). At 12-month postoperative follow-up, although there was no patient with score 1 in new technique group, 5 patients was reported in Norfolk group. Meanwhile, 4 patients in new technique group and 7 patients in Norfolk group gave score 2 to the results. There were 16 patients in new technique group compared to 8 patients in Norfolk group who believed that, the results were acceptable. The difference was statistically significant regarding the acceptable results between two groups (*p*<0.001).

At 6-month postoperative follow-up, one assessor scored 1, 4 assessors scored 2, and 15 assessors scored 3 to the results of Norfolk technique. Whereas, for new technique, one assessor scored 2, and 19 assessors scored 3. There was no significant difference regarding acceptable results between two groups (*p*=0.12). At 12-month follow-up, 4 assessors scored 1, 5 assessors scored 2, and 11assessors scored 3 in Norfolk group. However, for new technique group, 2 assessors scored 2, and 18 assessors scored 3 to the results. The number of acceptable results was statistically higher in new technique group compared to Norfolk group, based on assessors’ scores (*p*=0.01). Table 1 shows the distribution of patients and assessors scores in new and Norfolk techniques groups. 

**Table 1:** Distribution of patients and assessors scores in new and Norfolk techniques groups

	**New technique group (n)**	**Norfolk technique group (n)**
Total number of patients	20	20
Patients̓ Score=1 after 6 months	0	1
Patients̓ Score=2 after 6 months	3	5
Patients̓ Score=3 after 6 months	17	14
Patients̓ Score=1 after 12 months	0	5
Patients̓ Score=2 after 12 months	4	7
Patients̓ Score=3 after 12 months	16	8
Assessors̓ Score=1 after 6 months	0	1
Assessors̓ Score=2 after 6 months	1	4
Assessors̓ Score=3 after 6 months	19	15
Assessors̓ Score=1 after 12 months	0	4
Assessors̓ Score=2 after 12 months	2	5
Assessors̓ Score=3 after 12 months	18	11

**Fig. 4:**
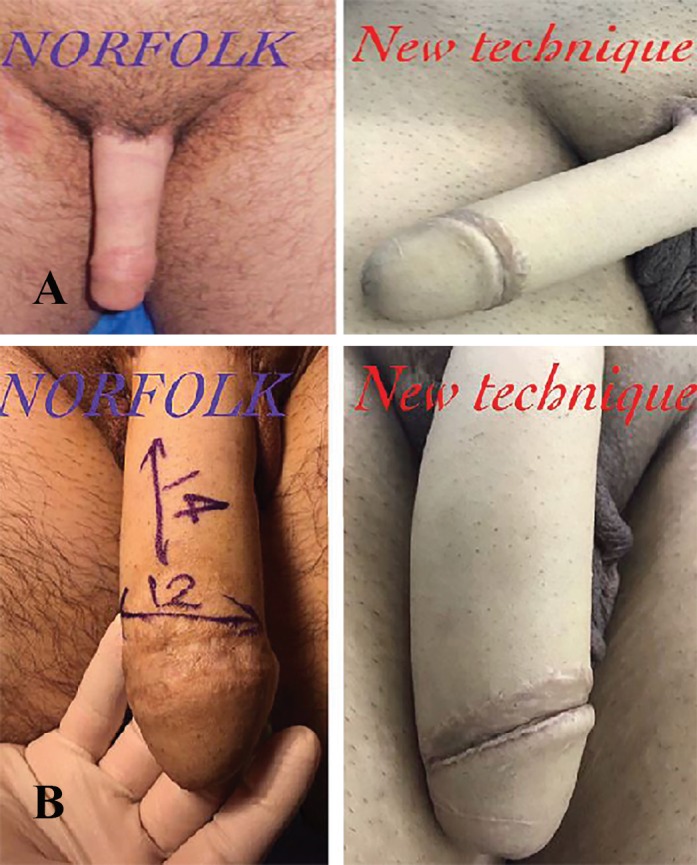
**A:** Results at 6 months post operation. **B: **Results at 12 months post operation

Almost 85% of the patients in new technique group were satisfied with the outcome of surgery and considered it as acceptable at 6-month follow-up; however, only 70% of patients in Norfolk group reported acceptable results, which was significantly lower than new technique group (Figure 4A). Similarly, at 12-month follow-up, 80 and 40% of the patients, respectively in new technique and Norfolk groups reported acceptable results ,which was also significantly higher (*p*<0.001) in new technique group (Figure 4B). Likewise, at 12-month postoperative follow-up, the assessors considered 90% of the results as acceptable in new technique group, which was remarkably significant compared to 55% of acceptable results in Norfolk group.

## DISCUSSION

Anatomically, the glans is cone-shaped with a circumferential ridge (the coronal ridge) at the base and a neck (coronal sulcus) proximal to the glans. The goal of glans sculpting in phalloplasty is creating a prominent coronal ridge and a constricted coronal sulcus (1). More importantly, it is crucial to provide the satisfaction for the patient and his partner, in terms of not only shape and size of the glance, but also regarding the psychological aspects. In this study, 40 patients were enrolled, which, to the best of our knowledge was the largest sample size in the literature. 

The patients were assigned to two different surgical techniques groups, and were followed up for 12 months post operation to be evaluated in terms of the results. This study is one the few studies in which the long-term results of new coronaplasty technique were objectively evaluated and accurately compared with Norfolk technique as current surgical method. There are many techniques available for glans sculpting; however, some of these techniques are no longer applied in practice. For instance, in 1957, Munawar introduced his method of glans sculpting; in which a circumferential skin flap was raised distally about 5 mm in width and folded back on itself to create a coronal ridge (2). 

In 1978, Puckette and Montie used several diamond skin excisions to create coronal ridge (3). In 1984, Chang and Hwang used two-tongue skin flaps in their free forearm phalloplasty to close the tip of the neoglans (1). In 1984, KAO *et al.* used autogenous cartilage and free skin flaps from the forearm based on the radial artery, for phalloplasty and coronaplasty (4). In 1989, sheng *et al.* used an expander in a small area of hairless skin on the lateral arm and reconstructed the neo phallus, and the Stanford group used a transverse elliptical skin flap to shape the neoglans (5). In 1995, Wing and Gilbert used a distal island flap to create a glans (6).

Recent novel techniques have revolutionized the glansplasty. For example, in **Gottleib design**, labial tissue is used for improved pigmentation of the glans. In **Horton technique**, a circumferentially deepithelializing skin flap is raised at the level of the proposed coronal ridge, which is then rolled-up, and is sutured to the free edges of the flap at its own base; thus, forming a ridge (7). In Hage method, a reasonable, non-circumcised appearing phallus was achieved in 7 patients, but Norfolk method should be applied when a circumcised appearance of the phallus is desired (8). In 2009, Salgado *et al.* used palmaris longus tendon for coronaplasty in a patient (9). In 2018, Sommeling *et al.* used two separate full-thickness skin grafts, the first at the raw undersurface of the flap, and the second at the flap’s donor site (10). 

In this study, the results of a new surgical technique were presented for glance sculpturing, in which natural-appearance penis was reconstructed with more permanent results using a medium thickness skin graft and dorsal aspect coverage of distally raised flap with mattress sutures. This new technique showed remarkably better results relative to the usual technique for glans sculpting in transsexual patients. Moreover, it has the ability to be easily applied along with ALT/RFFF flaps in both immediate and delayed situations. Applying this new technique, permanent results such as coronal ridge, constricted coronal sulcus, aesthetically and naturally-appearing glans could be achieved. Therefore, it is strongly recommended as an alternative technique for glance sculpturing in current practice. 

## CONFLICT OF INTEREST

The authors declare no conflict of interest.
